# Signaling via the Interleukin-10 Receptor Attenuates Cardiac Hypertrophy in Mice During Pressure Overload, but not Isoproterenol Infusion

**DOI:** 10.3389/fphar.2020.559220

**Published:** 2020-10-30

**Authors:** Nicholas Stafford, Farryah Assrafally, Sukhpal Prehar, Min Zi, Ana M. De Morais, Arfa Maqsood, Elizabeth J. Cartwright, Werner Mueller, Delvac Oceandy

**Affiliations:** ^1^ Division of Cardiovascular Sciences, Faculty of Biology, Medicine and Health, The University of Manchester, Manchester, United Kingdom; ^2^ Manchester Academic Health Science Centre, The University of Manchester, Manchester, United Kingdom; ^3^ School of Biological Sciences, The University of Manchester, Manchester, United Kingdom

**Keywords:** interleukin-10, cardiac hypertrophy, signaling pathway, inflammation, heart failure

## Abstract

Inflammation plays a key role during cardiac hypertrophy and the development of heart failure. Interleukin-10 (IL-10) is a major anti-inflammatory cytokine that is expressed in the heart and may play a crucial role in cardiac remodeling. Based on the evidence that IL-10 potentially reduces pathological hypertrophy, it was hypothesized that signaling via the IL-10 receptor (IL10R) in the heart produces a protective role in reducing cardiac hypertrophy. The aim of this study was to investigate the effects of the ablation of *Il-10-r1* gene during pathological cardiac hypertrophy in mice. We found that IL-10R1 gene silencing in cultured cardiomyocytes diminished the anti-hypertrophic effect of Il-10 in TNF-α induced hypertrophy model. We then analyzed mice deficient in the *Il-10-r1* gene (IL-10R1^-/-^ mice) and subjected them to transverse aortic constriction or isoproterenol infusion to induce pathological hypertrophy. In response to transverse aortic constriction for 2 weeks, IL-10R1^-/-^ mice displayed a significant increase in the hypertrophic response as indicated by heart weight/body weight ratio, which was accompanied by significant increases in cardiomyocyte surface area and interstitial fibrosis. In contrast, there was no difference in hypertrophic response to isoproterenol infusion (10 days) between the knockout and control groups. Analysis of cardiac function using echocardiography and invasive hemodynamic studies did not show any difference between the WT and IL-10R1^-/-^ groups, most likely due to the short term nature of the models. In conclusion, our data shows that signaling via the IL-10 receptor may produce protective effects against pressure overload-induced hypertrophy but not against β-adrenergic stimuli in the heart. Our data supports previous evidence that signaling modulated by IL-10 and its receptor may become a potential target to control pathological cardiac hypertrophy.

## Introduction

Heart failure remains one of the primary causes of morbidity and mortality across the global spectrum of cardiovascular disease. Despite current advances in the therapeutic approach, prognosis of this disease remains poor and its prevalence is rising (Mozaffarian et al., 2015; Taylor et al., 2017). Understanding the pathophysiology of heart failure is essential for the development of effective and efficient new therapeutic approaches.

The involvement of pro-inflammatory cytokines in HF was first reported three decades ago, when elevated TNF-α was found in the serum of HF patients (Levine et al., 1990). Since then, growing bodies of evidence have indicated the key roles of inflammatory mediators in the development of HF (reviewed in (Adamo et al., 2020)). Almost all primary myocardial injuries such as pressure overload and ischemia can trigger activation of the immune response. In addition, molecules that are released by damaged myocytes, for example heat shock proteins, fibronectins and reactive oxygen species, may activate residential macrophages and other immune cells, which eventually triggers the release of inflammatory cytokines (Mann, 2015).

It is widely accepted that cytokines are involved in the pathogenesis of heart failure, not only by affecting the inflammatory response but also by directly affecting cardiomyocytes and other cells in the heart such as fibroblasts and vascular cells. This is underlined by the fact that cardiomyocytes and other non-inflammatory cells in the heart are also producing cytokines and expressing their receptors under stress (Yoshida et al., 2005). Evidence has shown that pro-inflammatory cytokines contribute significantly to the pathogenesis of heart failure, in particular in modulating left ventricular remodeling and myocardial contractility. For example, cardiac overexpression of TNF-α in mice induces cardiac hypertrophy and reduces contractility (Kubota et al., 1997), whereas ablation of TNF-α attenuates cardiac hypertrophy, inflammation, apoptosis and fibrosis following aortic constriction (Sun et al., 2007).

In contrast to the knowledge on the role of pro-inflammatory cytokines in the heart, less is known about the roles of anti-inflammatory molecules in the context of cardiac hypertrophy and heart failure. One of the best characterized anti-inflammatory cytokines in the heart is the interleukin-10 (IL-10). IL-10 is mainly produced by T-cells and is able to modulate both acute and chronic inflammation (reviewed in (Ouyang and O'Garra, 2019)). The main targets of IL-10’s actions are inflammatory cells expressing the IL-10 receptor (IL-10R) such as macrophages/monocytes, dendritic cells, T and B cells (Ouyang and O'Garra, 2019). However, expression of the IL-10 receptor in cardiomyocytes is also reported (Yoshida et al., 2005), suggesting that IL-10 may have a direct effect on these cells.

Several prior studies have shown beneficial effects of IL-10 treatment in pathological conditions. Observations in isolated cardiomyocytes have shown beneficial effects of IL-10 in inhibiting TNF-α induced apoptosis and oxidative stress (Dhingra et al., 2007; Dhingra et al., 2009). Likewise, treatment with IL-10 produces beneficial effects in animal models of myocardial infarction and pressure overload hypertrophy (Krishnamurthy et al., 2009; Verma et al., 2012; Jung et al., 2017). In addition, involvement of IL-10 in the clinical setting has been underlined by the finding that serum IL-10 is elevated in HF patients (Lindberg et al., 2008). However, the precise characterization of IL-10’s effects in the cardiomyocytes and in the heart, and whether it exerts these effects via activation of its receptor are not fully understood. Therefore, we will address this question in the present project by investigating the effects of IL-10R1 gene ablation in the setting of pathological cardiac hypertrophy.

## Materials and Methods

### Isolation of Neonatal Rat Cardiomyocytes

Primary neonatal rat cardiomyocytes (NRCM) were derived from 1- to 3- days old Sprague Dawley rat neonates. The neonates were sacrificed by cervical dislocation and the hearts were removed and put into filter-sterilized ADS solution pH 7.35 (116 mM NaCl, 20 mM HEPES, 1 mM NaH2PO4, 5.5 mM glucose, 5.5 mM KCl and 1 mM MgSO4). The hearts were sliced into small pieces and then digested in ADS solution containing 0.6 mg/ml collagenase A (Roche) and 0.6 mg/ml pancreatin (Sigma) in a shaking 37°C water bath for 7 min. Digested cells were collected and the process was repeated a further seven times. Cells were pooled and centrifuged at 1,200 r.p.m. for 5 min. The pellet was then resuspended in 40 ml of pre-plating medium (68% DMEM, 17% M199, 10% horse serum, 5% FBS and 2.5 mg/ml amphotericin B). The cardiac fibroblasts were removed by plating cells in 10 mm culture dishes for 1 h to allow them to adhere, and then retrieving cardiomyocytes from the media supernatant. NRCM were then plated into 6-well BD Falcon Primaria tissue culture plates (for expression analysis) or on laminin coated coverslips in 24-well plates (for cellular hypertrophy analysis). Plating medium was similar to pre-plating medium, with the addition of 1 mM BrdU (5-bromo-2-deoxyuridine). From the second day onward, cardiomyocytes were cultured in maintenance medium (80% DMEM and 20% Medium 199, 1% FBS, 2.5 mg/mlamphotericin B and 1 mM BrdU).

### *In vitro* Experiments

For expression analysis, NRCM were plated in 6 well plates at a density of 1 × 10^6^ cells per well. After 24 h, cells were infected with adenovirus expressing either control or IL-10R shRNA driven by the U6 promoter. The shRNA constructs were obtained from SABiosciences (Qiagen). The U6-shRNA fragments were cloned into the pENTR11 and then recombined to pAd-PL-DEST (Gateway system, Invitrogen) to obtain the adenovirus construct. Adenovirus were generated in HEK293 cells using a standard protocol.

For the cellular hypertrophy models, cells were plated in 24-well plates onto laminin coated coverslips (Sigma, 10 μg/ml) at a density of 5 × 10^4^ cells per well. After 24 h, cells were infected with Ad-shRNA control or Ad-shIL-10R in serum-free maintenance medium. To induce hypertrophy, 10 ng/ml TNF-α (Sigma) or 1 µM isoproterenol (Sigma) were added for 48 h, while the IL-10 treatment group received 20 ng/ml recombinant IL-10 (Peprotech) for the same time period. After 48 h cells were fixed and permeabilised with 4% PFA and 0.1% Triton-X solution, respectively. Cells were stained with anti-α-actinin antibody (Sigma) and DAPI to visualize the cardiomyocytes and nuclei, respectively, and imaged using an Olympus BX51 fluorescent microscope at 20× magnification. A minimum of 100 cells per treatment group were measured per independent experiment using ImageJ software (NIH).

### Animal Model

We used mice with ubiquitous ablation of the IL-10R1 gene. Generation of these mice has been described in a previous publication (Pils et al., 2010). In brief, the IL-10R1 allele was mutated by the insertion of two loxP sites flanking exon 1 and the promotor region of IL-10R1. To generate mice with systemic deletion of the IL-10R1 gene, the IL-10R1flox/flox mice were crossed with transgenic mice expressing Cre recombinase in early development (the (K14-Cre,B6.D2-Tg(KRT14-cre)1Cgn) strain)(Hafner et al., 2004). The human keratin 14 promoter is active in driving transgene expression in the epidermal tissues. When female mice carry this K14-Cre transgene the promoter is active in their oocytes (Hafner et al., 2004). Consequently, breeding of IL-10R1flox/flox mice with female K14-Cre mice will result in systemic deletion of the IL-10R1 gene (Pils et al., 2010).

All experiments using animals were performed in accordance with the United Kingdom Animals (Scientific Procedures) Act 1986 and were approved by the University of Manchester Ethics Committee.

### Transverse Aortic Banding

Pressure overload was induced by constricting the transverse aorta using a method as previously described (Mohamed et al., 2016). Briefly, 8–12 weeks old mice were induced with 5% isoflurane and intubated orally, and thereafter maintained at 3% isoflurane during surgery with mechanical ventilation. The chest was opened via minithoracotomy and the aortic arch exposed. Constriction was performed by tying a 7–0 silk suture around a 27-gauge needle overlying the arch, between the origin of the brachiocephalic trunk and left common carotid artery. Sham surgery followed the same procedure, but the suture was passed around the aorta and withdrawn without tying. The chest was then sutured shut and mice administered with 0.1 mg/kg BW buprenorphine as analgesia before recovery at 30°C and return to normal housing. After 14 days final analyses were performed, and mice were sacrificed via an approved schedule one method before heart excision and storage at −80°C.

### Isoproterenol-Induced Hypertrophy

To generate a model of isoproterenol-induced hypertrophy, mice received a dose of 10 mg/kg BW per day isoproterenol (Sigma) or saline control for 10 days via a subcutaneously implanted osmotic minipump (Alzet). For minipump implantation, mice were anesthetized with 3% isoflurane. A small horizontal incision was made through the dermal layers on the dorsal surface of the mouse to create a small pocket into which minipumps were implanted. The skin was sutured shut, following which mice received analgesia (0.1 mg/kg BW buprenorphine) and were allowed to recover at 30°C before return to normal housing.

### Echocardiography Analysis

For transthoracic echocardiography, mice were anesthetized by IP injection of tribromoethanol (250 mg/kg BW, Sigma), following which hearts were imaged in the two-dimensional short-axis view with an Acuson Sequoia C256 ultrasound system fitted with a 14-MHz transducer (Siemens). Chamber dimensions and wall thicknesses were measured in systole and diastole from M-mode images taken in the short-axis view at the level of the papillary muscle using the leading-edge method over a minimum period of three cardiac cycles.

### Invasive Hemodynamic Analysis

Hemodynamic analysis was performed as described in our previous publication (Mohamed et al., 2016). Following administration of anesthetic (tribromoethanol, 250 mg/kg BW by IP injection), a midline cervical incision was made and the sternohyoid muscles retracted. The exposed right carotid artery was tied at its bifurcation and occluded proximally, allowing an incision to be made with minimal blood loss. Through this incision, a 1.4 F pressure–volume catheter (SPR-839, Millar Instruments) was inserted and fed through the ascending aorta into the left ventricle. A PowerLab system (Millar Instruments) was used to record LV pressure–volume changes once traces had stabilized. The maximum and minimum rates of left ventricular pressure change, dP/dtmax and dP/dtmin, respectively, were used to assess cardiac function in systole and diastole using Millar’s PVAN software.

### Histology

A transverse section through the ventricles of approximately 1mm in thickness, was cut from excised hearts and fixed in 4% PFA overnight. Sections were then dehydrated overnight in a Leica automated tissue processor and embedded in paraffin wax, before sectioning at 5 µM using a rotary microtome (Leica 2255). Masson’s trichrome and H and E staining were performed to quantify interstitial fibrosis and cell size, respectively, using standard protocols. Whole sections were imaged using a 3D Histech Panoramic slide scanner in the University of Manchester bioimaging facility. Cell size measurements were acquired from a minimum of 100 cells per heart, and total LV interstitial fibrosis was recorded.

### Western Blot

NRCM were lyzed in 100 µl RIPA buffer (containing 1% IGEPAL CA-630, 0.5% sodium deoxycholate, 0.1% SDS, 0.5 mM phenylmethylsulphonyl fluoride, 500 ng/ml Leupeptin, 1 mg/ml Aprotinin and 2.5 mg/ml Pepstatin A). Western blot analysis was conducted using standard protocols. Proteins were separated by SDS-PAGE and transferred to PVDF membranes (Millipore). Membranes were blocked in 3% BSA and incubated with primary antibodies against IL-10R1 (Santa Cruz), phosphorylated STAT3 or total STAT3 (Cell Signaling) overnight, followed by HRP-linked anti-rabbit secondary antibody (Cell Signaling) for 2 h. Proteins were visualized using enhanced chemiluminescence (GE Healthcare) on a ChemiDoc XRS Imaging System (Biorad). Membranes were then incubated with β-actin or GAPDH antibody (abcam) as loading control.

### Statistical Analysis

Data was analyzed using Microsoft Excel and GraphPad Prism. Data is expressed as mean ± s.e.m. Student’s t-test or one-way ANOVA followed by post-hoc adjustment for multiple comparisons were used where appropriate. The probability level for statistical significance was set at *p* < 0.05.

## Results

### Interleukin-10R1 Gene Silencing in Cardiomyocytes

**FIGURE 1:**
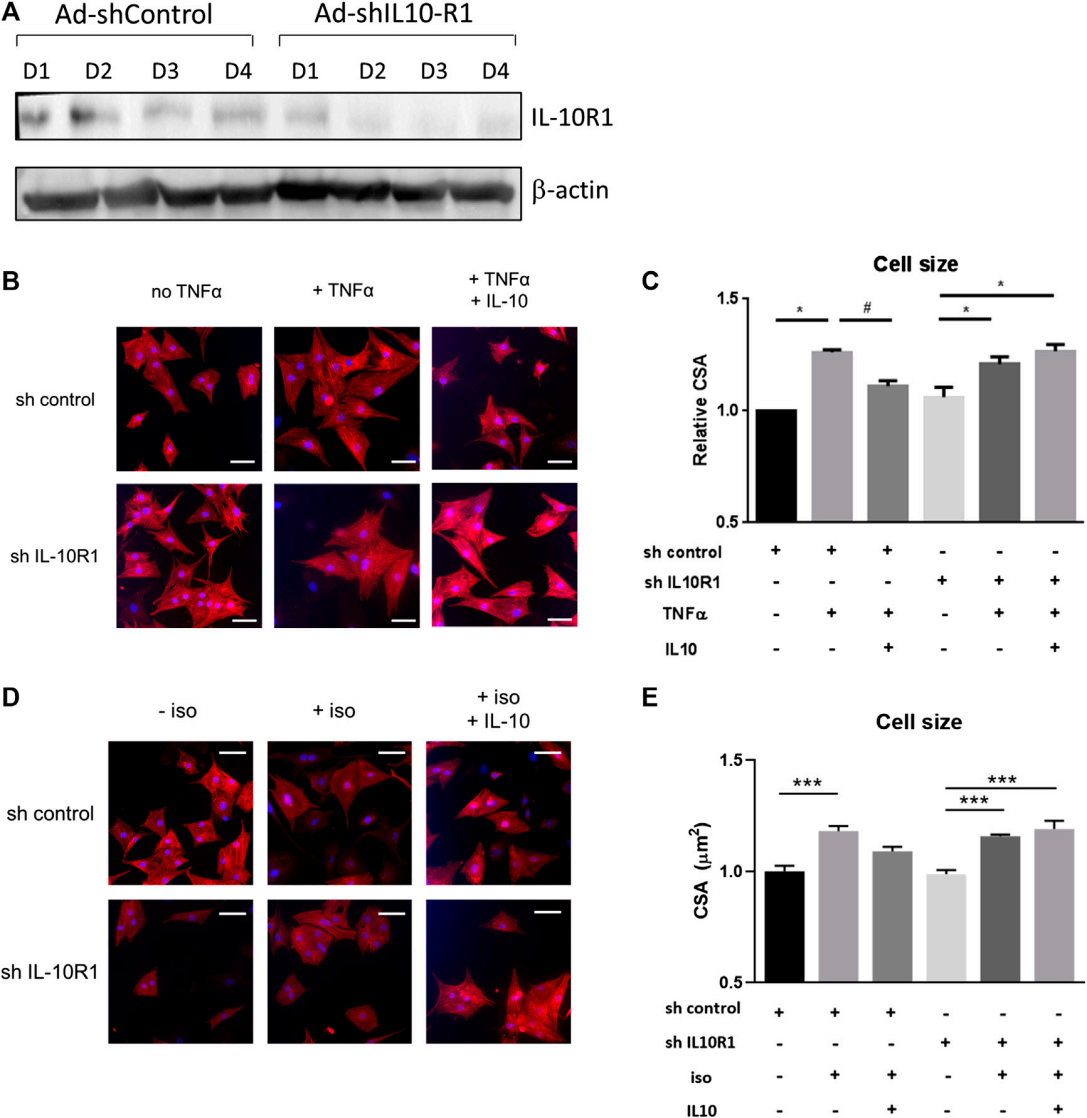
Effects of IL-10 treatment and IL-10R1 gene silencing in TNF-α-induced cardiomyocyte hypertrophy. **(A)** Western blot analysis showing the expression of IL-10R1 in NRCM treated with adenovirus expressing either control shRNA or shRNA for IL-10R1 for 1–4 days. **(B)** Representative images of NRCM stained with α-actinin (red) and DAPI (blue) expressing either shRNA control or shRNA IL-10R1 after treatment with TNF-α (10 ng/ml) or with or without addition of IL-10 (20 ng/ml) for 48 h. Scale bar = 25 μM **(C)** Quantification of cell surface area (N = 3 independent experiments, with a minimum of n = 100 cells per experiment,*p < 0.05 vs. no TNF, #*p* < 0.05 vs. TNF without IL-10 **(D)** NRCM treated with 1 µM isoproterenol for 48 h ± IL-10. Scale bar = 25 µM and **(E)** quantification of myocyte cross-sectional area (N = 4 independent experiments, minimum 50 cells each, ***p < 0.001).

It has been reported that IL-10 treatment produces beneficial effects in controlling adverse cardiac remodeling following pressure overload or acute myocardial infarction in rodents(Krishnamurthy et al., 2009; Verma et al., 2012; Jung et al., 2017). However, the signaling pathways modulated by IL-10 in the cardiomyocytes are not fully understood. To investigate whether IL-10 exerts its function through activation of the IL-10 receptor (IL-10R), we generated adenovirus expressing shRNA to inhibit the expression of the IL-10R1 gene encoding the main sub-unit of the receptor, in isolated neonatal rat cardiomyocytes (NRCM). Marked reduction of IL-10R1 expression was observed at day 2–3 following adenovirus treatment (Figure 1A) providing evidence of successful silencing of this gene.

### Signaling via Interleukin-10R1 Is Necessary to Inhibit Hypertrophy in Isolated Cardiomyocytes

In immune cells, IL-10 mediated-signalling represses the pro-inflammatory signals of cytokines such as TNF-α and IL-6 by inducing the STAT3-mediated expression of anti-inflammatory gene SOCS3 (Cassatella et al., 1999; Williams et al., 2004). In cardiomyocytes the pro-inflammatory cytokines including TNF-α and IL-6 are known to induce hypertrophy (Kubota et al., 1997; Melendez et al., 2010). Based on this knowledge we performed experiments to investigate whether IL-10 and its receptor are involved in mediating TNF-α induced hypertrophy. We tested this hypothesis in primary rat neonatal cardiomyocytes (NRCM). In these cells TNF-α treatment induced cardiomyocyte hypertrophy by 26% as indicated by cell size measurement. As expected, co-treatment with IL-10 abolished the pro-hypertrophic effect of TNF-α (Figures 1B,C). Interestingly in IL-10R1 deficient cardiomyocytes, although TNF-α on its own was able to induce hypertrophy, treatment with IL-10 failed to reduce the hypertrophic effect (Figures 1B,C). This finding suggests that IL-10 mediated signaling in cardiomyocytes is important in repressing the hypertrophic effect of TNF-α, and sufficient expression of the IL-10R1 gene is required for this effect.

We then examined whether signaling via the IL-10R1 also affected hypertrophy induced by the β-adrenergic agonist isoproterenol. NRCM displayed significantly increased cell size following 48 h of isoproterenol treatment, while co-treatment with IL-10 reduced the hypertrophic response (Figures 1D,E). As with TNF-α treatment, isoproterenol also induced hypertrophy in IL-10R1 deficient cardiomyocytes, which was not affected upon co-treatment with IL-10 (Figures 1D,E). These findings suggest that signaling via the IL-10 receptor may modulate cardiac hypertrophy induced by multiple agonists.

### Genetic Ablation of the Interleukin-10R1 Gene Exaggerates Pathological Hypertrophy in Mice

**FIGURE 2:**
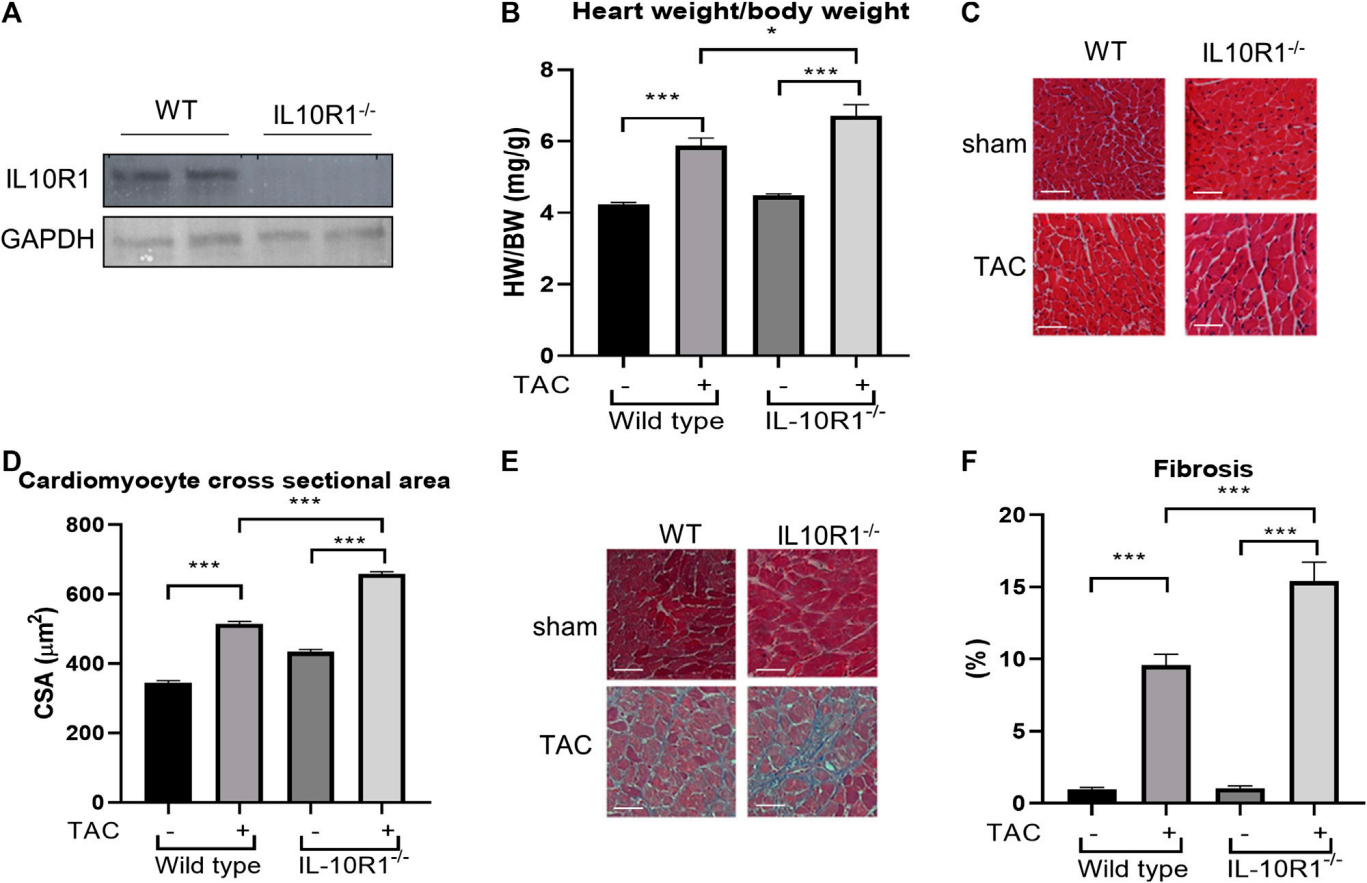
Pressure overload-induced hypertrophy model in IL-10R1^-/-^ mice. **(A)** Representative Western blot of total heart extracts from IL-10R1^-/-^ mice and WT littermates showing complete ablation of IL-10R1 expression in the knockout mice. **(B)** Analysis of heart weight/body weight ratio (HW/BW) of WT and IL-10R1^-/-^ mice following transverse aortic constriction (TAC) for 2 weeks. IL-10R1^-/-^ mice showed a significant increase in hypertrophic response compared to WT littermates (WT-sham, n = 13; WT-TAC, n = 19; KO-sham, n = 11; KO-TAC, n = 12, *p < 0.05, ***p < 0.001). **(C)** Histological sections of heart tissues stained with hematoxylin and eosin and **(D)** quantification of cardiomyocyte cross-sectional area showed enhanced cardiomyocyte size in IL-10R1^-/-^ mice after TAC. **(E)** Masson’s trichrome staining and **(F)** quantification of fibrotic area indicated significantly higher fibrosis in IL-10R1^-/-^ mice (WT-sham, n = 7; WT-TAC, n = 8; KO-sham, n = 8; KO-TAC, n = 7) Scale bars = 50 µM.

Results from the *in vitro* model prompted us to question if IL-10R1 plays a key role in mediating cardiac hypertrophy in an *in vivo* model. To address this question we analyzed mice with global ablation of the IL-10R1 gene (IL-10R1^-/-^). The generation of this strain was described previously (Pils et al., 2010). To confirm deletion of IL-10R1 in the heart, Western blot analysis was performed. Expression of IL-10R1 was completely ablated in total heart extracts of IL-10R1^-/-^ mice (Figure 2A). To investigate the pathologic hypertrophic response we subjected the IL-10R1^-/-^ and wild type (WT) littermates to transverse aortic constriction (TAC) for 2 weeks. Global ablation of IL-10R1 significantly enhanced the hypertrophic response to pressure overload as indicated by heart weight/body weight (HW/BW) ratio. We observed a 48% increase in HW/BW ratio in IL-10^-/-^ mice compared to 29% increase in WT littermates (Figure 2b). Consistently, measurement of cardiomyocyte cross-sectional area from histological sections showed a marked increase in cardiomyocyte size in the IL-10R1^-/-^ TAC group compared to WT TAC (Figures 2C,D).

### Interleukin-10R1^-/-^ Mice Display More Fibrosis in the Heart After Transverse Aortic Constriction

Cardiac fibrosis is an important detrimental feature of the heart’s response to pathological stimuli. To assess the level of fibrosis in the heart we stained heart tissue sections with Masson’s trichrome staining. Analysis of fibrotic area as depicted in Figures 2E,F revealed a significantly higher fibrosis level in IL-10R1^-/-^ mice after TAC compared to the WT-TAC group.

### Echocardiography Analysis to Assess Cardiac Morphology and Function

**FIGURE 3:**
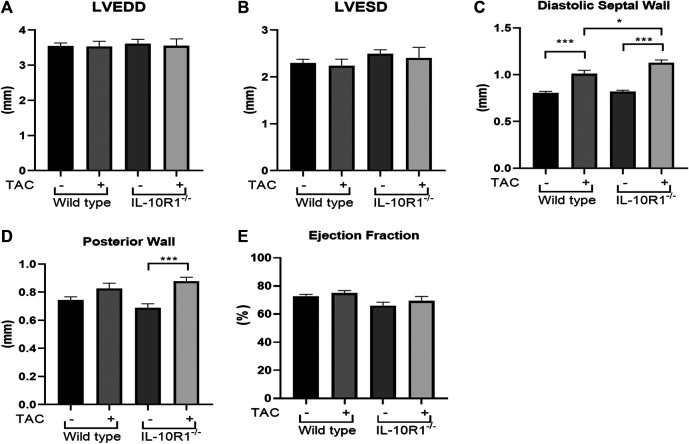
Echocardiography analysis of IL-10R1^-/-^ mice following TAC. **(A)** Left ventricular end diastolic dimension (LVEDD) and **(B)** Left ventricular end systolic dimension (LVESD) were not different between IL-10R1^-/-^ mice and WT controls basally and after TAC. **(C)** Ventricular septal wall thickness at diastole was bigger in IL-10R1^-/-^ mice after TAC, while **(D)** posterior wall thickness did not differ between these genotypes. **(E)** There was no significant difference in ejection fraction between all experimental groups (WT-sham, n = 13; WT-TAC, n = 15; KO-sham, n = 11; KO-TAC, n = 12,*p < 0.05,***p < 0.001).

To further analyze cardiac morphology and function of IL-10R1^-/-^ mice following pressure overload we carried out echocardiography analysis at 2 weeks after TAC. We found that there was no difference in the left ventricular chamber dimension at both diastole and systole (LVEDD and LVESD, Figures 3A,B). As expected, the ventricular wall thickness was significantly elevated in both groups after TAC. However, we found that the IL-10R1^-/-^ mice exhibited significantly greater septal wall thickness. Also, although there was no statistically significant difference in the posterior wall thickness between WT vs. IL-10R1^-/-^ mice, the increase in posterior wall thickness seemed to be more apparent in the knockout mice compared to WT (Figures 3C,D).

We also analyzed ejection fraction from the echocardiography data to assess if the pathological damage affected cardiac function. We found that there was no difference in ejection fraction between IL-10R1^-/-^ vs. WT mice after TAC (Figure 3E).

### Invasive Hemodynamic Analysis to Assess Heart Function

**FIGURE 4:**
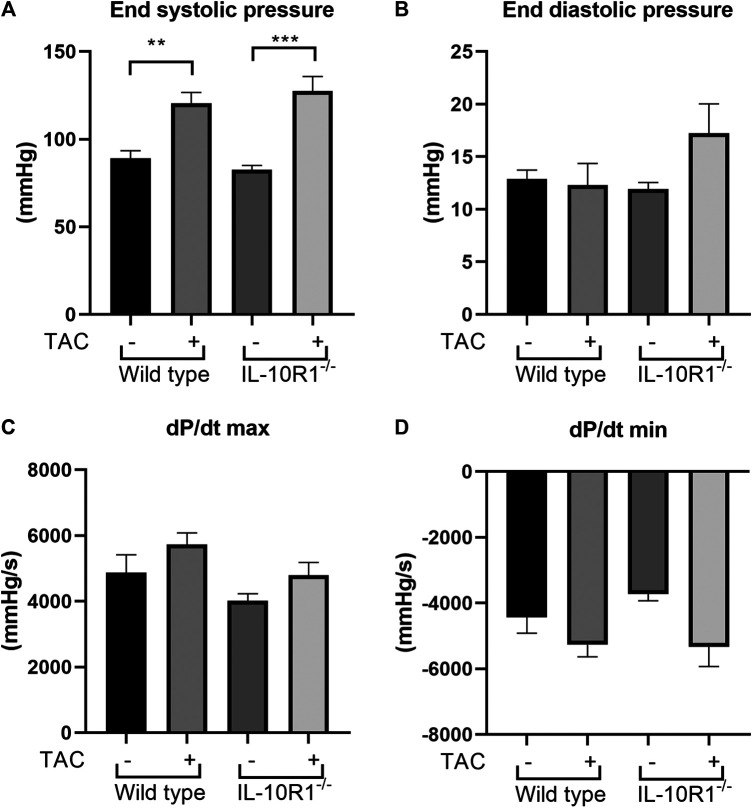
Invasive hemodynamics assessment using micro-catheter. **(A)** End systolic ventricular pressure was significantly increased in both genotypes following TAC, however there was no difference between IL-10R1^-/-^ mice vs. WT. **(B)** There was no statistically significant difference in end systolic pressure **(C)** dP/dt max and **(D)** dP/dt min in all experimental groups(WT-sham, n = 13; WT-TAC, n = 15; KO-sham, n = 11; KO-TAC, n = 12, **p < 0.01, ***p < 0.001).

Following echocardiography measurement, we conducted analysis to evaluate cardiac hemodynamic functions by inserting a pressure-volume catheter into the left ventricle. As shown in Figure 4A, the end-systolic pressure was markedly elevated in both WT and IL-10R1 knockout mice after TAC, indicating successful induction of pressure-overload in both groups. The fact that there was no difference in cardiac end-systolic pressure between these groups confirmed that a comparable degree of overload was applied to each genotype. The end-diastolic pressure was not different between the groups (Figure 4B).

We then measured the rate of developed pressure during systole (dP/dt max) and the rate of reduced pressure during diastole (dP/dt min). We found no difference of dP/dt max and dP/dt min in all groups tested (Figures 4C,D). This data was consistent with the ejection fraction analysis showing that there was no change in contractility after 2 weeks of pressure overload.

### Isoproterenol-Induced Hypertrophy Model in Interleukin-10R1^-/-^ Mice

**FIGURE 5:**
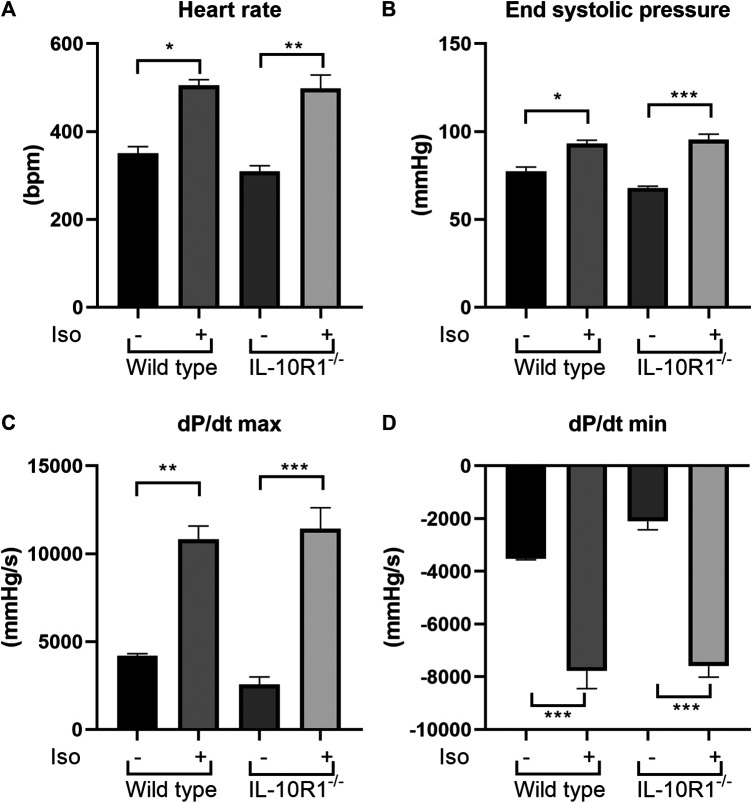
Effects of isoproterenol infusion in IL-10R1^-/-^ mice and WT littermates. **(A)** Heart rate and **(B)** end systolic pressure were significantly increased in both genotypes following isoproterenol infusion (10 mg/kg BW/day) for 10 days. Consistently, **(C)** the rate of developed ventricular pressure (dP/dt max) and **(D)** the rate of ventricular relaxation (dP/dt max) were increased following isoproterenol treatment. However, there was no significant difference between IL-10R1^-/-^ mice vs. WT controls (WT-vehicle, n = 3; WT-Iso, n = 6; KO-vehicle, n = 3; KO-Iso, n = 7, *p < 0.05,**p < 0.01,***p < 0.001).

To investigate whether the effects of IL-10R1 genetic ablation also occur in a different model of cardiac hypertrophy, we established β-adrenergic mediated hypertrophy by stimulation with isoproterenol. We implanted osmotic minipumps subcutaneously in mice to enable continuous infusion of isoproterenol (10 mg/kg BW/day) for 10 days. As expected, isoproterenol infusion markedly increased heart rate and systolic pressure compared to vehicle-treated groups (Figures 5A,B). As a consequence, the rate of developed pressure (dP/dt max) and relaxation (dP/dt min) were dramatically enhanced following isoproterenol treatment (Figures 5C,D). However, we did not observe an altered hemodynamic response to isoproterenol stimulation between IL-10R1^-/-^ and WT mice as indicated by these parameters.

**FIGURE 6:**
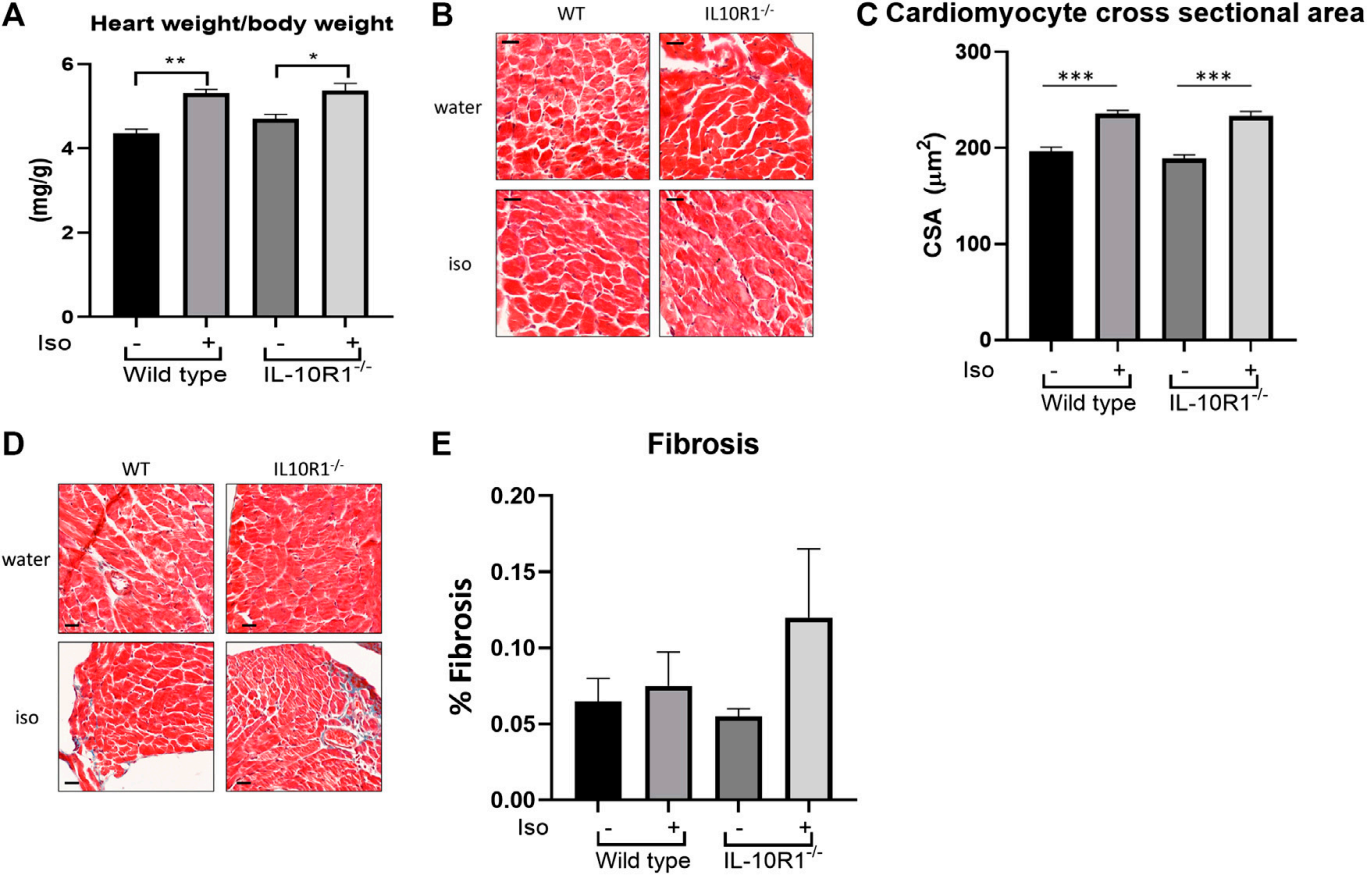
Hypertrophic and fibrotic response in isoproterenol model. **(A)** Although there was significantly increased HW/BW ratio following isoproterenol infusion, there was no significant difference between IL-10R1^-/-^ and WT mice (WT-vehicle,n = 3; WT-Iso, n = 6; KO-vehicle, n = 3; KO-Iso, n = 7,*p < 0.05,**p < 0.01). **(B)** Histological sections of heart tissues stained with hematoxylin and eosin and **(C)** quantification of cardiomyocyte cross-sectional area **(D)** Masson’s trichrome staining and **(E)** quantification of fibrotic area following isoproterenol infusion (WT-vehicle, n = 3; WT-Iso, n = 4; KO-vehicle, n = 3; KO-Iso, n = 3,***p < 0.001) Scale bars = 25 µM.

Furthermore, we found a significant enlargement of heart size following isoproterenol in both groups; interestingly however, there was no significant difference in HW/BW ratio between IL-10R1^-/-^ vs. WT mice suggesting a comparable hypertrophic response to beta adrenergic stimulation (Figure 6A). These results were corroborated by histological measurement of cardiomyocyte cross sectional area, which was increased significantly by isoproterenol, but did not differ when comparing IL-10R1^-/-^ and WT hearts (Figures 6B,C). We also assessed levels of fibrosis in Masson’s trichrome stained sections following β-adrenergic stimulation, but did not find that isoproterenol induced significant levels of interstitial fibrosis in either group (Figures 6D,E).

**FIGURE 7:**
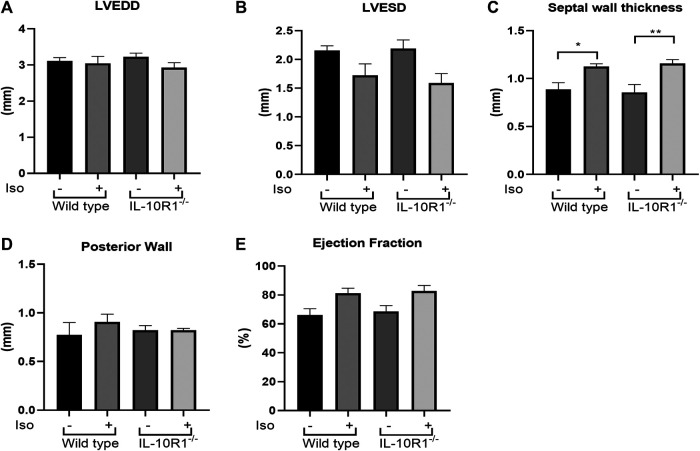
Echocardiography analysis of IL-10R1^-/-^ mice in isoproterenol model. **(A)** Echocardiography assessment revealed that there was no difference in LVEDD and **(B)** LVESD among experimental groups. **(C)** Septal wall thickness was augmented in both groups after isoproterenol treatment but there was no difference between genotypes. **(D)** No difference in posterior wall thickness and **(E)** Ejection fraction among experimental groups at the end of isoproterenol treatment (WT-vehicle, n = 3; WT-Iso, n = 6; KO-vehicle, n = 3; KO-Iso, n = 7,*p < 0.05,**p < 0.01).

Detailed echocardiographic analysis revealed that isoproterenol did not alter left ventricular dimensions but increased ventricular wall thickness in both genotypes. However, consistent with the HW/BW data there was no significant difference between WT and IL-10R1^-/-^ groups (Figures 7A–D). Similarly, we did not find any difference in posterior wall thickness or ejection fraction between IL-10R1^-/-^ vs. WT group after induction with isoproterenol (Figure 7E). Together, the data showed that there was no difference in the hypertrophic and hemodynamic response to beta-adrenergic stimulation between IL-10R1^-/-^ mice compared to WT controls.

### The Inflammatory Response in Transverse Aortic Constriction and Isoproterenol Treated Mice

**FIGURE 8:**
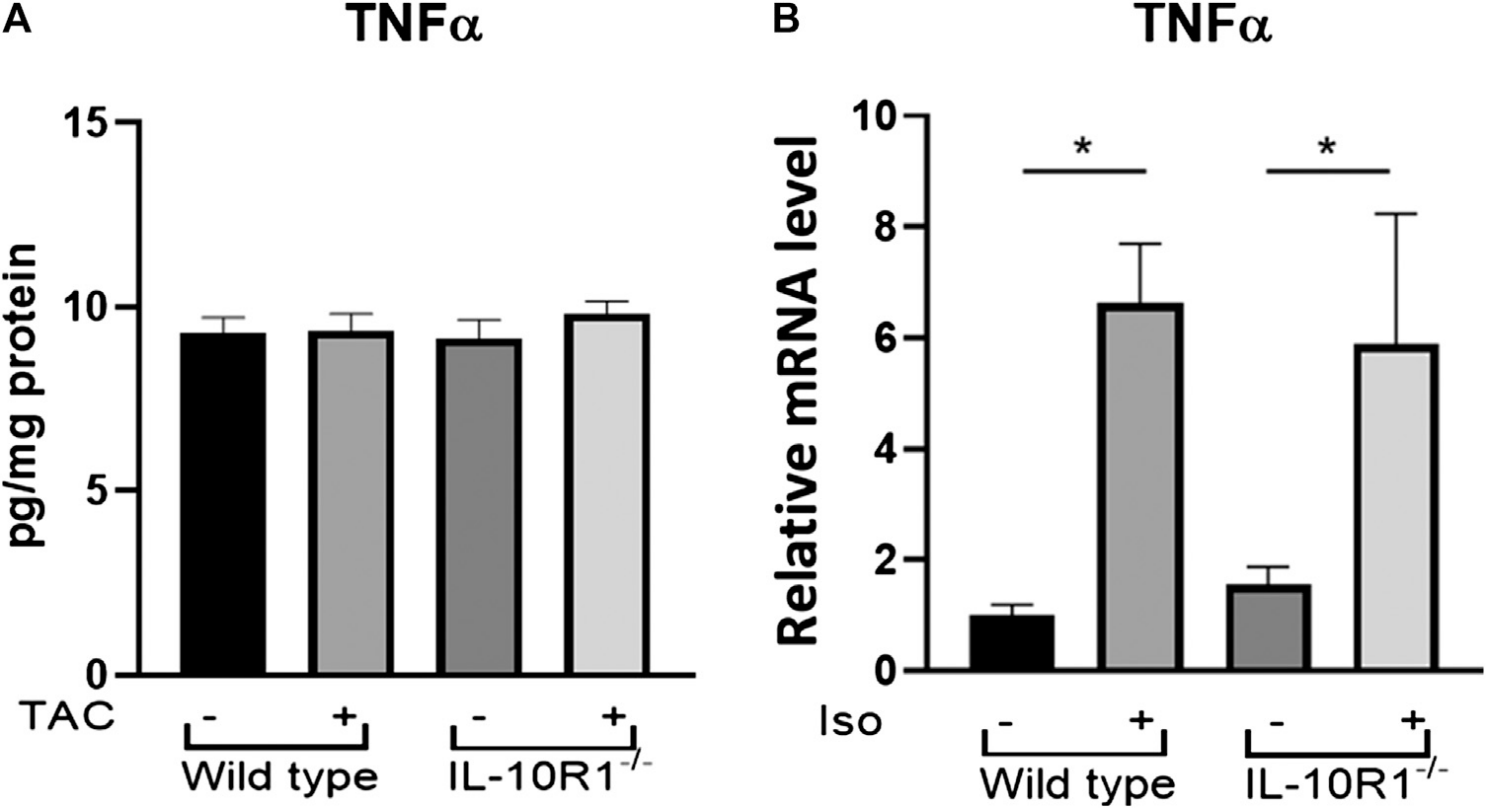
TNF-α levels in TAC and isoproterenol models. **(A)** Analysis of TNF-α levels in heart tissue extracts from WT and IL-10R1^-/-^ mice following TAC as measured by ELISA (n = 5) **(B)** Cardiac TNF-α mRNA expression relative to β-actin as loading control did not differ between isoproterenol treated WT and IL-10R1^-/-^ mice (WT-vehicle, n = 3; WT-Iso, n = 4; KO-vehicle, n = 3; KO-Iso, n = 3,*p < 0.05).

In order to examine whether the different responses to TAC and isoproterenol treatment in IL-10R1^-/-^ mice could be due to altered levels of inflammation, we examined the level of pro-inflammatory cytokine TNF-α in each model. Performing ELISA on extracts from pressure-overloaded heart tissue revealed no significant difference in TNF-α concentration among sham and TAC hearts, nor IL-10R1^-/-^ and WT hearts 2 weeks post-TAC (Figure 8A). We also examined expression of TNF-α in isoproterenol-infused heart tissue by qPCR. In contrast to the TAC model, we found isoproterenol to increase TNF-α expression compared to water controls, although we did not see a difference in levels between IL-10R1^-/-^ and WT mice (Figure 8B). This indicates that the inflammatory response may have been different in the two models used in this study.

### Interleukin-10R1 Deficiency Prevents IL-10-Induced STAT3 Activation

**FIGURE 9:**
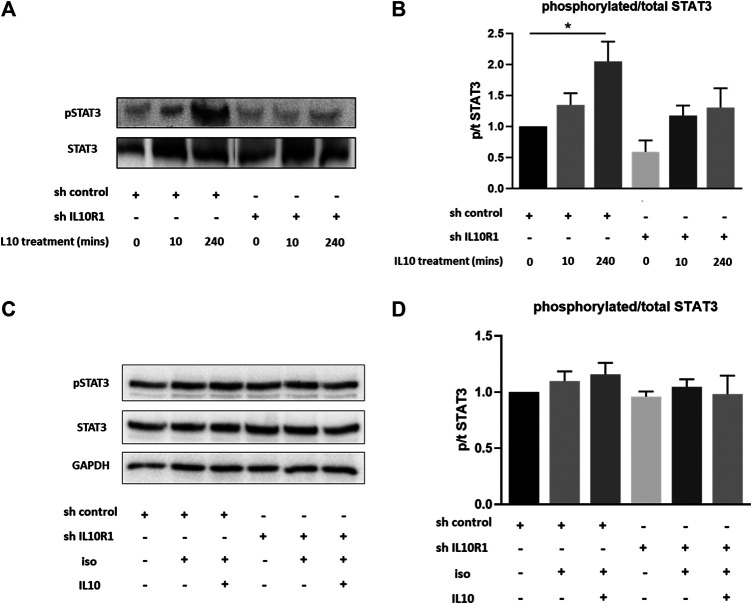
IL-10 induced activation of STAT3. **(A)** Representative western blot showing STAT3 phosphorylation in shRNA control or shRNA IL-10R1 NRCM basally, and following the addition of 20 ng/ml IL-10 for 10 min or 4 h **(B)** Ratio of phosphorylated:total STAT3 (N = 5,*p < 0.05) **(C)** Representative western blot showing STAT3 phosphorylation in shRNA control or shRNA IL-10R1 NRCM following the addition of 1 µM isoproterenol with or without 20 ng/ml IL-10, along with loading control GAPDH **(D)** Ratio of phosphorylated:total STAT3 (N = 3).

Previous studies have shown IL-10 to exert protective effects in response to TAC and isoproterenol via activation of STAT3, and subsequent suppression of p38 and NF-kB signaling (Verma et al., 2012). We therefore examined activation of STAT3 in IL-10R1 deficient NRCM. Upon stimulation with IL-10, control myocytes displayed significantly increased phosphorylation, and hence activation, of STAT3 after 4 h (Figures 9A,B). This effect was abolished in IL-10R1 deficient NRCM. We then examined whether isoproterenol treatment affected IL-10 induced STAT3 activation. Interestingly, we found that isoproterenol treated NRCM did not exhibit activation of STAT3 upon addition of IL-10 (Figures 9C,D). These results indicate that IL-10 induced activation of STAT3 is dependent upon intact signaling via the IL-10 receptor, while other pathways may also be involved during β-adrenergic stimulation.

## Discussion

Our findings suggest that intact IL-10 receptor mediated signaling in the heart is required to control the extent of myocardial hypertrophy following pressure overload stimulation. Interestingly, our data demonstrate that ablation of the IL-10 receptor did not alter hypertrophy in response to β-adrenergic stimulation. IL-10 is known as a powerful negative regulator of the inflammatory response (Mosser and Zhang, 2008). Thus, our results confirm previous findings that an uncontrolled inflammatory response in the heart might cause an excessive hypertrophic response to pathological stimuli.

Cardiac hypertrophy is known as an initial adaptive compensatory response to pathological stimuli (Burchfield et al., 2013). The myocardial tissue produces numerous proteins in response to stress mainly to limit the extent of tissue damage, to induce tissue repair and to preserve function (Doroudgar and Glembotski, 2011). Among the molecules increased in pathologic hearts, inflammatory mediators appear to have essential roles since they may modulate cardiac function and contractile remodeling as well as altering endothelial function (Prabhu and Frangogiannis, 2016). For example, pro-inflammatory cytokines which are known to be elevated in human failing hearts, such as TNF-α, IL-1β and IL-6, negatively regulate cardiac contractility (Pagani et al., 1992; Janssen et al., 2005; Van Tassell et al., 2013). In addition, these cytokines are also involved in mediating cardiac remodeling as evidenced by the finding that genetic deletion of the TNF-α gene leads to a reduction in TAC-induced hypertrophy (Sun et al., 2007), whereas infusion of IL-6 in rat induces cardiac hypertrophy (Melendez et al., 2010). These findings support the idea that excessive release of pro-inflammatory cytokines is detrimental to the heart.

Conversely, evidence has indicated protective roles of anti-inflammatory cytokines in the heart. IL-10, which is a powerful anti-inflammatory cytokine, reduces the extent of hypertrophy in response to pressure overload and angiotensin II (Verma et al., 2012; Kishore et al., 2015), improves cardiac function and inhibits adverse remodeling post-myocardial infarction (Stumpf et al., 2008; Krishnamurthy et al., 2009; Jung et al., 2017). Importantly, in patients with severe heart failure the level of serum IL-10 is significantly reduced (Stumpf et al., 2003). Furthermore, survivors of acute myocardial infarction with higher serum IL-10 levels are less likely to develop heart failure (Domínguez Rodríguez et al., 2005).

It is believed that IL-10 exerts its protective role by suppressing the production of inflammatory mediators through activation of the STAT3 signaling pathway (Williams et al., 2004). Components of the innate immune system such as monocytes, macrophages and neutrophils are thought to be the main target of IL-10 (Pils et al., 2010). Following binding and activation of the IL-10 receptor in these cells, STAT3 will be recruited and phosphorylated, triggering expression of STAT3 target genes, notably the suppressor of cytokine signaling 3 (SOCS3) (Niemand et al., 2003). The latter is a strong inhibitor of IL-6 activity by interfering with gp130 signaling (Babon et al., 2014).

In this study we sought to address an important question as to how IL-10 reduced cardiac hypertrophy. Our *in vitro* model demonstrated that signaling through the IL-10 receptor in cardiomyocytes was required to exert the anti-hypertrophic response of IL-10 treatment, since deficiency of the major sub-unit of the receptor IL-10R1, abolished the anti-hypertrophic effect of IL-10 upon TNF-α and isoproterenol treatment. Indeed, it remains to be elucidated if this process is also important in the regulation of the cardiomyocyte response to other stimuli, such as α-adrenergic agonists or angiotensin. Nevertheless, the data prompted us to question if ablation of the Il-10R1 gene *in vivo* would alter the cardiac hypertrophic response. We generated two models of hypertrophy, i.e. TAC-induced and isoproterenol-induced hypertrophy. In our hands we found that the receptor deficiency only affected the TAC-induced hypertrophic response, but not the response to isoproterenol stimulation. This is not to say that IL-10 signaling is not capable of protecting the heart from chronic β-adrenergic stimulation. A previous study has shown that IL-10 knockout mice exhibit a slightly worse phenotype to isoproterenol stimulation, albeit at a higher dose and for a longer period than the one used in our study, and that IL-10 treatment was protective in this model (Verma et al., 2012). Since IL-10 likely works by reducing the effects of pro-inflammatory factors, it is possible that in the TAC-model and at higher doses of isoproterenol, a different inflammatory response occurs compared to the one in our isoproterenol model. In this study we examined TNF-α levels in heart tissue following 2 weeks TAC and 10 days isoproterenol, and in fact found little evidence of inflammation in the TAC model at this time point. Further analysis is needed to understand the exact role that inflammatory cytokines are playing in each model. For example, it is thought that inflammation is more active in the first days of TAC-induced hypertrophy, which also coincides with the greatest extent of hypertrophic growth (Bacmeister et al., 2019).

While we show here that signaling via IL-10R1 is important in regulating cardiac hypertrophy and fibrosis after TAC, we did not find that ablation of the receptor translated to any functional deterioration after 14 days pressure overload. It would be interesting to see if left ventricular function declined in IL-10R1 ablated mice in a more chronic TAC model. Indeed, IL-10 knockout mice have been shown to exhibit reduced fractional shortening and ejection fraction 28 days post TAC, which was not apparent at 14 days (Verma et al., 2017).

It would also be interesting to further investigate the cellular and molecular mechanisms through which signaling via the IL-10 receptor acts during pressure overload. Our *in vitro* data suggests that activation of IL-10 signaling directly in cardiomyocytes can negatively regulate hypertrophy; however our *in vivo* TAC model utilized mice with global ablation of IL10R1 and therefore cannot rule out further involvement of IL-10 signaling in other cell types such as macrophages or lymphocytes. In order to investigate the precise actions of IL-10 signaling in each cell type tissue specific knockouts would be required.

Furthermore, it will be important to further explore the molecular mechanisms involved in the regulation of pressure overload and isoproterenol induced-hypertrophy and fibrosis downstream of the receptor. Previous studies have shown IL-10 signaling to exert protective effects during pressure overload via activation of STAT3 signaling, and subsequent suppression of p38, NF-κB and TGF-β-Smad2/3 signaling (Verma et al., 2012; Verma et al., 2017). On the other hand chronic angiotensin II infusion has been shown to also activate Akt, p38 and NF-κB pathways in IL-10 knockout mice (Kwon et al., 2016). Here we confirm that IL-10 can activate STAT3 in cardiomyocytes, and that the IL-10R1 is required for this. We also found that isoproterenol treatment prevented this IL-10 induced activation. Further studies will be needed to elucidate the full signaling pathways downstream of the receptor involved in regulating the hypertrophic response to each stimuli.

Overall, this study adds to the growing body of evidence suggesting that IL-10 based therapy may be a promising avenue to develop treatments for a number of cardiac pathologies associated with heart failure, including those induced by pressure overload (Verma et al., 2012; Verma et al., 2017), myocardial infarction (Krishnamurthy et al., 2009), high fat diet (Kondo et al., 2018) and angiotensin (Kishore et al., 2015). Anti-inflammatory based-therapies may offer a more attractive target compared to pro-inflammatory cytokine antagonists such as anti-TNF-α treatment, which have failed to show benefits in clinical trials in heart failure patients, likely due to TNF-α’s pleiotropic nature (Chung et al., 2003; Mann et al., 2004). However, while IL-10 based therapy is attractive, further work will be required in order to perfect the stability and delivery of the peptide to the heart, as trials for its use in diseases such as rheumatoid arthritis and Crohn’s disease have found only modest improvements (Mosser and Zhang, 2008). The development of stable recombinant IL-10 will be the key to future therapeutic advances.

## Data Availability Statement

The raw data supporting the conclusions of this article will be made available by the authors, without undue reservation.

## Author Contributions

NS, performed experiments, analyzed data, wrote manuscript; FA, performed experiments, analyzed data; SP and MZ, performed *in vivo* experiments; AMDM and AM, performed *in vitro* experiments; EJC, designed and supervised *in vivo* experiments; WM conceived scientific idea; DO, conceived scientific idea, designed experiments, analyzed data, supervised the whole project, wrote manuscript.

## Funding

This study was supported by British Heart Foundation (BHF) Project Grants (PG/16/77/32400, PG/17/78/33304, PG/18/40/33767), Medical Research Council (MRC) Research Grant (MR/P015816/1) to DO and a BHF Project Grant PG/11/23/28801 jointly held by DO and WM

## Conflict of Interest

The authors declare that the research was conducted in the absence of any commercial or financial relationships that could be construed as a potential conflict of interest.
